# The long and winding road to diagnosis: diagnostic delays and healthcare utilization in patients with CFT-confirmed INOCA

**DOI:** 10.3389/fcvm.2026.1924728

**Published:** 2026-10-07

**Authors:** Angela Wang, Rose Huang, Yvonne Shea, Harmony R. Reynolds, Nathaniel R. Smilowitz, Anaïs Hausvater

**Affiliations:** 1 Department of Internal Medicine, NYU Grossman School of Medicine, New York, NY, United States; 2 Graduate Medical Education, NYU Grossman School of Medicine, New York, NY, United States; 3 Center for Women’s Cardiovascular Health, Leon H. Charney Division of Cardiology, NYU Langone Health, New York, NY, United States; 4 Section of Cardiology, Department of Medicine, VA NY Harbor Health Care System, New York, NY, United States

**Keywords:** clinical science, coronary physiology, coronary vasospasm, INOCA, ischemic cardiac disease

## Abstract

**Background:**

Patients with Ischemia and No Obstructive Coronary Arteries (INOCA) experience recurrent angina, report reduced quality of life, and are at increased risk for major adverse cardiovascular events. Unfortunately, INOCA is often unrecognized and can be difficult to diagnose.

**Methods:**

We included patients with angina and INOCA diagnosed by invasive coronary function testing (CFT) and characterized their interactions with the healthcare system. Data on initial symptom onset, frequency and type of healthcare encounters, specialty referrals, diagnostic testing (including non-invasive and invasive cardiac testing, pulmonary, GI, and musculoskeletal evaluation), and medication trials for angina were abstracted from the electronic medical record.

**Results:**

We included 91 patients with INOCA who underwent CFT for determination of endotype (36% with coronary microvascular dysfunction, 35% with vasomotor disorders, 29% mixed endotype). The median time from first anginal symptom to diagnosis was 3.2 years (IQR 0.8–7.3 years), with 450 presentations to the healthcare system for angina prior to CFT. Among healthcare presentations, 72% occurred in an outpatient setting and 28% occurred in the emergency department. Each patient had a median of 3 clinic visits and 1 ED visit. A total of 225 referrals to 6 different specialties occurred, with 18% of patients seen by ≥3 cardiologists prior to diagnosis. There were 441 diagnostic tests performed for angina [median 4 (IQR 3–7) per patient], 14% of which were non-cardiac studies [median 0 (IQR 0–1) per patient]. Patients trialed a median of 2 (1–3) medications prior to diagnosis.

**Conclusion:**

Many patients with INOCA experience prolonged diagnostic journeys, marked by repeated healthcare encounters, multiple specialists’ visits, high testing burden, and multiple trials of medication. These findings underscore the need for earlier consideration of INOCA in the assessment of angina and more efficient diagnostic strategies.

## Introduction

Ischemia with no obstructive coronary arteries (INOCA) is a syndrome characterized by symptoms and signs of ischemia without obstructive coronary artery disease (<50% stenosis) that disproportionately affects women (1, 2). INOCA is associated with adverse outcomes: compared with age- and race-matched controls without angina or cardiovascular disease, patients with INOCA have up to 3-fold increase in risk of myocardial infarction and hospitalization for heart failure, stroke, or cardiac mortality (3, 4). Angina frequency in INOCA is comparable to patients with obstructive coronary artery disease (CAD), and these symptoms are associated with decreased quality of life (5, 6). Patients with INOCA experience higher levels of depression and anxiety, which may be partially driven by diagnostic uncertainty (7, 8).

INOCA includes various mechanisms or “endotypes” including coronary microvascular dysfunction, coronary vasospasm, endothelial dysfunction, microvascular spasm, and mixed (9). Invasive coronary function testing (CFT) or non-invasive imaging modalities such as stress cardiac magnetic resonance imaging (MRI) or cardiac positron emission tomography (PET) scan are used to identify INOCA endotypes (10). Diagnosis of INOCA can be challenging as traditional diagnostic testing validated for the workup of obstructive CAD is often normal (11). CFT-confirmed diagnosis can improve angina severity and quality of life among patients with INOCA, but it remains underutilized and only available at a handful of specialized centers (10–14). Most patients report delays in diagnosis and a high burden of diagnostic testing that is frequently inconclusive and poses a financial burden (9, 11, 15).

We sought to evaluate the diagnostic journey for patients with INOCA from symptom onset to CFT, including diagnostic testing, encounters with the healthcare system, and trials of anti-anginal medications.

## Methods

### Study design and participants

In a single center, retrospective cohort study, we identified adults with INOCA who underwent invasive CFT at NYU Langone Health between July 2018 and March 2026. Patients were eligible if they had 1) stable anginal symptoms and/or ischemia on stress testing and 2) < 50% stenosis in any major epicardial coronary artery determined during invasive coronary angiography. This amounted to a total of 154 patients who underwent CFT. Of these, 58 patients had normal CFT and were deemed to have non-cardiac chest pain. Of the remaining 96 patients, 5 patients were excluded due to incomplete documentation (Figure 1).

**Figure 1:**
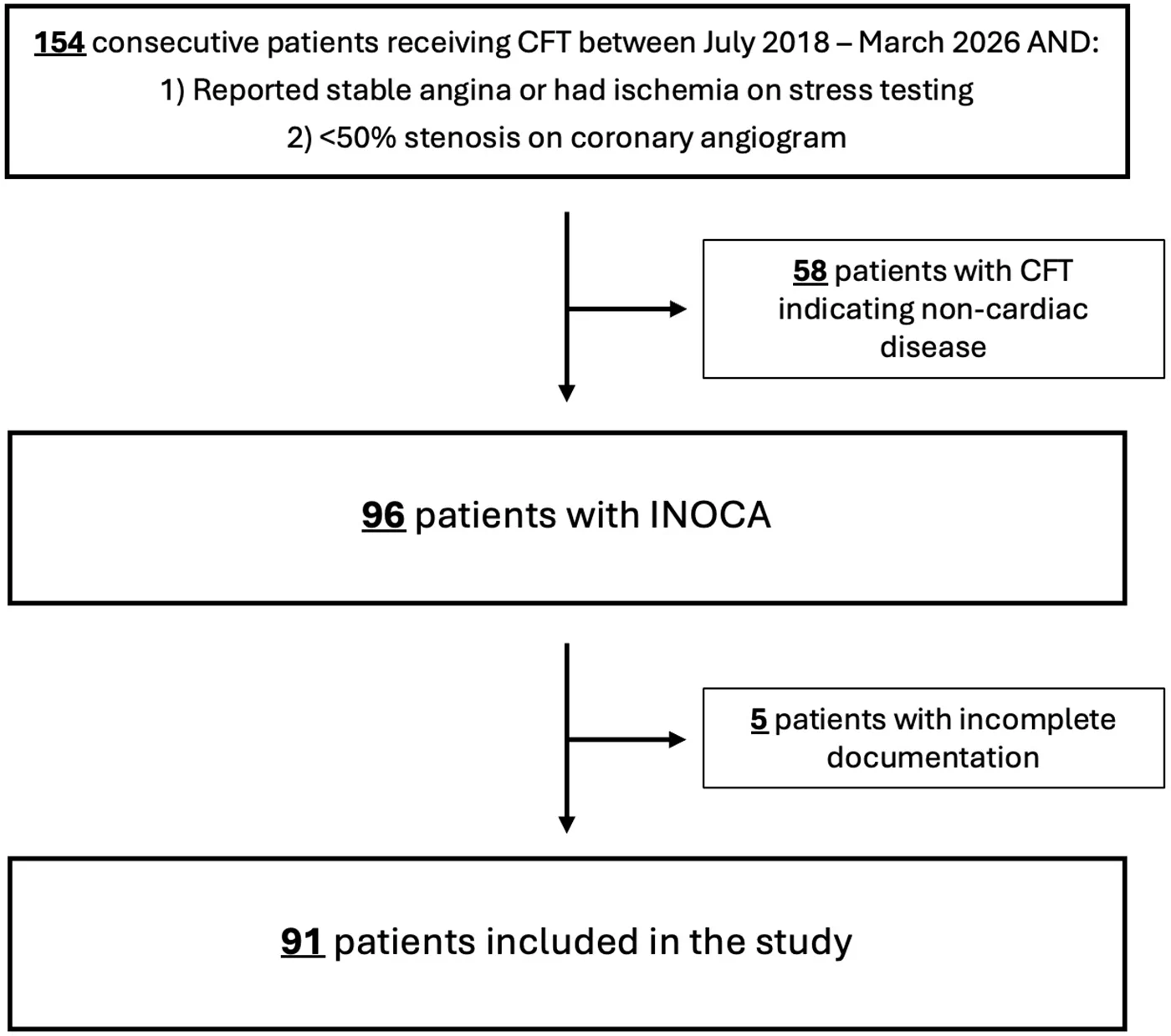
Patient cohort selection.

### Data collection

Data were divided between two physician reviewers and manually extracted from each participant's electronic medical record. For ascertainment of symptoms, all available notes were reviewed including physician progress notes, emergency department documentation, and nursing notes. Additionally, the EMR was reviewed for medications, medical testing (including referral orders and imaging reports), and procedure reports. Angina was defined as any potentially ischemic symptom, including but not limited to chest pain or discomfort, jaw/arm pain, dyspnea, or dyspnea on exertion.

All terms potentially related to angina including “chest pain”, “chest discomfort”, “jaw/arm pain”, “dyspnea”, and “dyspnea on exertion” were searched in the chart for the earliest healthcare encounter with one of the above complaints. The clinical documentation was then reviewed for the first patient reported symptom onset. For notes that described patient first reported symptom onset “at the beginning of the month”, the first day of the month was documented. If these specifics were not documented, then the date of the clinic encounter was used as the approximate date of angina. Patients were excluded if documented angina dates were too vague, for example “Patient reported chest pain has been going on for years”. Participants were also excluded if they lacked adequate documentation to ascertain symptoms, if physician progress notes were not available to the study team, or if results of diagnostic testing were missing. Any cases in which timeline of anginal symptoms were unclear, a third investigator (AH) reviewed the patient's chart and adjudicated any inconsistencies.

Beginning from the date of the earliest reported anginal symptom, all subsequent documented encounters in the medical record were reviewed to characterize each patient's diagnostic trajectory. Invasive CFT-based diagnosis was considered the endpoint of the patient's diagnostic journey.

### Definition of INOCA and endotypes

All patients included in this study underwent coronary angiography and were determined to have <50% stenosis in any major epicardial artery. Our study included 3 categories of INOCA endotypes based on CFT: coronary microvascular dysfunction (CMD), vasomotor disorders, and mixed (16). CMD was defined based on a coronary flow reserve (CFR) < 2.5 or index of microcirculatory resistance (IMR) ≥ 25. Vasomotor disorders included patients with epicardial coronary spasm, symptomatic endothelial dysfunction, and microvascular spasm. Epicardial coronary spasm was defined as >90% narrowing in response to acetylcholine coronary reactivity testing, with reproduction of nitrate responsive anginal symptoms and/or ECG changes. Symptomatic endothelial dysfunction was defined as >0% and ≤90% narrowing of epicardial coronary arteries and/or <50% change in coronary blood flow with acetylcholine accompanied with patient report of chest pain or an anginal equivalent. Microvascular spasm was defined as angina as well as ischemic ECG changes with acetylcholine without visible epicardial spasm (17). We chose to group patients with epicardial coronary spasm, symptomatic endothelial dysfunction, and microvascular spasm as one group given our small sample sizes for the purposes of statistical analysis. Patients classified as mixed endotype satisfied the criteria for both CMD and vasomotor disorders.

### Aspects of diagnostic journey

Healthcare encounters were categorized as emergency department or ambulatory clinic visits. All visits with subspecialists (cardiologists, gastroenterologists, pulmonologists, rheumatologists, psychiatrists, etc.) to evaluate anginal symptoms were reviewed. Diagnostic tests reviewed for each patient included cardiac and non-cardiac testing: echocardiography, exercise and nuclear stress testing, invasive angiography, coronary computed tomography angiography (CTA), cardiac magnetic resonance imaging (MRI), Holter monitoring, cardiac positron emission tomography (PET), stress cardiac MRI, right heart catheterization, pulmonary function testing (PFT), chest CTA, endoscopy, spine MRI, abdominal computed tomography (CT), barium swallow study, esophageal manometry, and other studies. We counted all anti-anginal medications as well as non-cardiac medications that were trialed for angina. Patients were referred to CFT for diagnostic clarification, medication intolerance, and/or persistent angina despite medications.

### Statistical analysis

The distribution of continuous variables was assessed prior to analysis. Normally distributed continuous variables were compared across endotype groups (CMD, vasomotor disorders, and mixed) using one-way analysis of variance (ANOVA), whereas skewed continuous variables were compared using the Kruskal–Wallis test. Categorical variables were compared using the chi-square test or Fisher's exact test, as appropriate. A *p*-value <0.05 was considered statistically significant.

## Results

A total of 91 adults with INOCA met study eligibility criteria and were included in the analysis. Overall, the mean age was 59.8 ± 11.6 years and 82% were female. At the time of CFT, 36% were diagnosed with CMD, 35% had vasomotor disorders, and 29% had a mixed endotype (Table 1). Patients had a high prevalence of cardiovascular comorbidities, including hypertension (63%), hyperlipidemia (75%), diabetes mellitus (15%), and current or former tobacco use (40%). Eleven patients (12%) had at least one presentation to the emergency room where they were found to have a troponin elevation.

**Table 1:** Baseline demographics and clinical characteristics of patient cohort, stratified by INOCA endotype.

Characteristic	Overall, *n* = 91	CMD, *n* = 33	Vasomotor Disorders, *n* = 32	Mixed, *n* = 26	*P*-value
Age, years mean (std dev)	59.8 (11.6)	63.2 (11.7)	54.0 (11.4)	63.0 (8.50)	**0** **.** 0007*
Female, *n* (%)	75 (82)	25 (76)	29 (91)	21 (81)	0.28^†^
Race/Ethnicity, *n* (%)					0.372^†^
White	59 (65)	20 (61)	22 (69)	17 (65)	
Black	10 (11)	3 (9)	2 (6)	5 (19)	
Asian	4 (4)	1 (3)	1 (3)	2 (8)	
Other	18 (20)	9 (27)	7 (22)	2 (8)	
Hispanic (%)	23 (25)	12 (36)	8 (25)	3 (11)	0.093^†^
BMI, kg/m2, Median (IQR)	27.6 (23.9–31.3)	28.8 (25.5–32.1)	27.5 (23.4–32.1)	25.4 (23.2–28.5)	0.052^‡^
Hypertension, *n* (%)	57 (63)	28 (85)	12 (38)	17 (65)	**<0**.**001**^†^
Diabetes, *n* (%)	14 (15)	9 (27)	3 (9)	2 (8)	0.059^†^
Hyperlipidemia, *n* (%)	68 (75)	26 (79)	22 (69)	20 (77)	0.619^†^
Current/Former Smoker, *n* (%)	36 (40)	17 (52)	13 (41)	6 (23)	0.084^†^

*denotes Anova testing, ^†^denotes chi-square testing for categorical variables, ^‡^denotes Kruskal–Wallis test for variables with skew.

The median time from the first reported anginal symptom to CFT with a diagnosis of an INOCA endotype was 3.2 years (IQR 0.8- 7.3 years). Between the three endotypes, the median time to diagnosis was 1.6 years (IQR 0.3–5.5 years) in patients with CMD, 4.0 years (0.8–7.6 years) in patients with vasomotor disorders, and 4.4 years (1.8–7.8 years) in patients with a mixed endotype. These were not statistically significant (*p* = 0.12 via Kruskal–Wallis test), though the study was not powered for significance. There were 450 presentations to the healthcare system for angina prior to CFT. Among healthcare presentations, 324 (72%) encounters occurred in an outpatient setting and 126 (28%) occurred in the emergency department. There was a median of 3 (IQR 2–5) outpatient visits per patient. There was a median of 1 (IQR 0–2) emergency department visits per patient for angina prior to CFT.

Figure 2 provides a summary illustration of the INOCA diagnostic journey. A total of 225 referrals occurred. The most common specialty visits for anginal symptoms were to cardiologists, pulmonologists, gastroenterologists, and rheumatologists. All patients were seen by at least one cardiologist, 27% of patients were treated by a pulmonologist, 16% of patients saw a gastroenterologist, and 3% were cared for by a rheumatologist. Patients saw a median of 2 (IQR 1–2) cardiologists prior to CFT, although 18% of patients were seen by 3 or more cardiologists prior to diagnosis confirmation.

**Figure 2:**
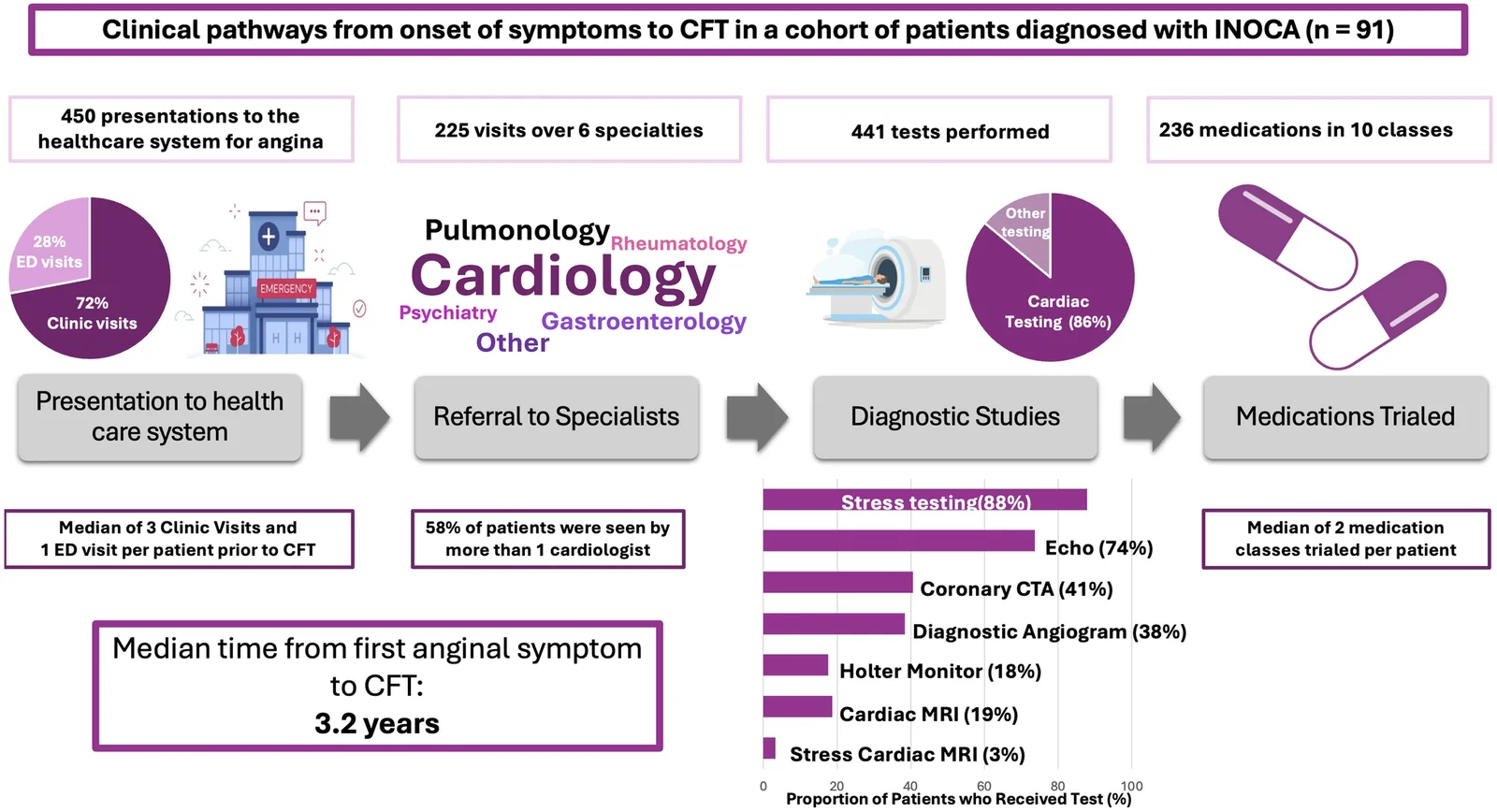
Summary of the diagnostic journey of patients who eventually received INOCA diagnosis confirmation with CFT. Percentages depict proportion of patients who received test.

Table 2 provides a summary of diagnostic testing among our cohort. There were 441 diagnostic tests performed for angina [median 4 (IQR 3–7) per patient], 14% of which were non-cardiac studies [median 0 (IQR 0–1) per patient]. Cardiac studies done to evaluate anginal symptoms included 117 echocardiograms (received by 74% of patient cohort), 83 exercise stress tests (70%), 54 nuclear stress tests (40%), 44 invasive angiograms without CFT (38%), 43 coronary CTAs (41%), 17 cardiac MRIs (19%), 16 Holter monitors (18%), 3 cardiac PET scans (3%), 3 stress cardiac MRI (3%), and 1 right heart catheterization (1%). Non-cardiac etiologies of anginal symptoms included 13 PFTs (received by 14% of patient cohort), 11 chest CTAs (12%), 10 endoscopies (11%), 3 spine MRIs (3%), 2 CT abdomens (2%), 1 barium swallow study (1%), and 1 esophageal manometry study (1%). There were 19 additional studies categorized as “other studies” received by 18% of the patient cohort. Other studies included sleep study, coronary calcium score, lower extremity venous and arterial duplex, CT chest, and carotid duplex.

**Table 2:** Diagnostic testing performed for patient cohort who eventually received diagnostic confirmation of INOCA with CFT. Other studies included sleep study, coronary calcium score, lower extremity venous and arterial duplex, CT chest, and carotid duplex.

Tests (*n* = 441)		Total number of tests performed	Number (%) of patients who received test
Cardiac testing, *n* = 381 (86% of total number of tests)	Echocardiogram	117	67 (74%)
	Exercise Stress Test	83	64 (70%)
	Nuclear Stress Test	54	36 (40%)
	Invasive Angiography	44	35 (38%)
	Coronary CTA	43	37 (41%)
	Cardiac MRI	17	17 (19%)
	Holter	16	16 (18%)
	PET test	3	3 (3%)
	Stress Cardiac MRI	3	3 (3%)
	Right Heart Catheterization	1	1 (1%)
Non-cardiac testing, *n* = 60 (14% of total number of tests)	Pulmonary Function Testing	13	13 (14%)
	Chest CTA	11	11 (12%)
	Endoscopy	10	10 (11%)
	Spine MRI	3	3 (3%)
	CT Abdomen	2	2 (2%)
	Barium Swallow Study	1	1 (1%)
	Esophageal Manometry	1	1 (1%)
	Other Studies	19	16 (18%)

Patients with INOCA trialed a median of 2 (IQR 1- 3) antianginal medication classes prior to CFT. Empiric medications included beta blockers (trialed by 31% of cohort), short acting nitrates (47%), long-acting nitrates (31%), calcium channel blockers (36%), and ranolazine (15%). Medications prescribed for alternate causes for anginal symptoms included proton pump inhibitors (11%), H2 blockers (5%), bronchodilators (5%), and selective serotonin reuptake inhibitors (4%).

## Discussion

In this cohort of patients with INOCA, our data illustrate the long and arduous diagnostic journey leading to CFT that characterizes this condition. The median time from first anginal symptom to receiving a definitive diagnosis was >3 years. Most patients were seen by 2 cardiologists before arriving at their diagnosis, more than half (55%) of patients had at least one ER visit for angina, and many were referred to additional specialists for diagnostic evaluation. Patients in our cohort underwent extensive cardiac and non-cardiac testing, and each patient trialed a median of 2 classes of medication prior to receiving a formal diagnosis through invasive CFT. Though there was a trend towards a difference in time to diagnosis by endotype, with patients with CMD having a shorter time to CFT, given the small numbers of our sample, these analyses are considered exploratory.

Our study results are consistent with prior studies demonstrating that patients with INOCA have frequent encounters with the healthcare system for persistent angina (1, 9). In our cohort, nearly 90% of patients had multiple outpatient clinic and emergency department visits prior to recognition of their diagnosis. Most patients had a prolonged course of both extensive workup and treatment trials prior to diagnosis confirmation with CFT.

We identified a recurring pattern of clinical care: once conventional assessments for obstructive CAD yielded normal results, anginal symptoms were attributed to non-cardiac chest pain, and patients were referred to subspecialists for non-cardiac diagnostic testing and trials of non-cardiac medications. This highlights the limited recognition of the clinical presentation of INOCA and awareness of the appropriate diagnostic evaluation. Limited experience in the care of INOCA is a global challenge. In a national survey of experienced Japanese cardiologists (60% interventional cardiologists), 54% of respondents had taken care of fewer than ten INOCA patients in their career (18). In an international survey of 457 interventional cardiologists, fewer than 27% of respondents performed CFT. Factors that led to increased uptake of CFT included ease of performing the procedure and knowledge of current guidelines, while financial and time constraints limited CFT adoption (19).

Timely and accurate diagnoses are critical in the management of INOCA because endotype-specific diagnosis and targeted medical therapy improve clinical outcomes (14, 20). The Coronary Microvascular Angina (CorMicA) trial was the first to demonstrate that invasive coronary testing with targeted therapy leads to superior quality of life and reductions in angina severity compared to usual care (14). Similar improvements in angina-related quality of life were seen in the Inclusive Invasive Physiological Assessment in Angina Syndromes—Angina with No Obstructive Coronary Artery Disease (ILIAS ANOCA) study (20). This raises the question as to whether all patients with angina and non-obstructive CAD should undergo CFT.

Invasive coronary function testing, stress cPET, and cMRI provide important insights into INOCA endotypes, but availability of these diagnostic modalities is limited to tertiary care referral centers (12, 19). Unfortunately, most patients with INOCA are not cared for at a specialized center, limiting access to appropriate diagnostics. Thus, many patients remain undiagnosed. This highlights the urgent need to develop and test lower cost, more scalable clinical diagnostics for INOCA endotypes.

Our study outlines important clinical implications of timely diagnosis of INOCA. We propose that patients who present with persistent angina despite non obstructive coronary arteries be offered either CFT or stress cPET or cMRI to further evaluate for INOCA. Doing so allows for confirmatory diagnosis and initiation of targeted medical therapies that can enhance the quality of life of patients while decreasing diagnostic uncertainty and reducing the burden of repeat healthcare encounters for angina.

### Limitations

This is a single-center retrospective observational study from a quaternary care referral center in patients who ultimately underwent CFT. This creates the potential for referral bias and may limit generalizability given many patients do not present to quaternary care institutions, do not receive diagnostic testing for INOCA endotypes, and remain undiagnosed. Consequently, our findings are limited to patients with access to specialized testing for INOCA which limits the generalizability to the larger INOCA population. Our cohort included eleven patients with myocardial infarction with non-obstructive coronary arteries (MINOCA). Having a troponin elevation on presentation may have led to expedited referral to a MINOCA/INOCA specialist. Data were abstracted from physician notes and clinical documentation with variable amounts of detail, and healthcare encounters in external health systems were only captured if the patients consented to share their records across institutions and external health systems used compatible electronic medical record. Events at hospitals with incompatible electronic medical records may not have been captured, potentially underestimating the true extent of healthcare utilization. Because symptom onset was retrospectively ascertained from clinical documentation, the estimated duration from symptom onset to diagnosis is subject to recall and documentation bias.

## Conclusions

Patients with INOCA experience prolonged and resource-intensive diagnostic pathways characterized by repeated healthcare encounters, extensive testing, and multiple medication trials prior to definitive diagnosis. Earlier recognition of INOCA and streamlined diagnostic strategies may reduce diagnostic delays, healthcare burden and improve quality of life for patients.

## Data Availability

The raw data supporting the conclusions of this article will be made available by the authors, without undue reservation.
